# Unraveling the Molecular Mechanism of Immunosenescence in *Drosophila*

**DOI:** 10.3390/ijms19092472

**Published:** 2018-08-21

**Authors:** Kyung-Jin Min, Marc Tatar

**Affiliations:** 1 Department of Biological Sciences, Inha University, Incheon 22212, Korea; 2 Department of Ecology and Evolutionary Biology, Brown University, Providence, RI 02912, USA

**Keywords:** innate immunity, aging, lifespan, *Drosophila*, antimicrobial peptide, juvenile hormone, 20-hyroxyecdysone, microbiota

## Abstract

A common feature of the aging process is a decline in immune system performance. Extensive research has sought to elucidate how changes in adaptive immunity contribute to aging and to provide evidence showing that changes in innate immunity have an important role in the overall decline of net immune function. *Drosophila* is an emerging model used to address questions related to immunosenescence via research that integrates its capacity for genetic dissection of aging with groundbreaking molecular biology related to innate immunity. Herein, we review information on the immunosenescence of *Drosophila* and suggest its possible mechanisms that involve changes in insulin/IGF(insulin-like growth factor)-1 signaling, hormones such as juvenile hormone and 20-hydroxyecdysone, and feedback system degeneration. Lastly, the emerging role of microbiota on the regulation of immunity and aging in *Drosophila* is discussed.

## 1. Introduction

Senescence is a process involving progressive conversion from healthy young adult into frail older ones. A common feature of it is a decline in immune performance. Increased age is accompanied by reduced capacity to thwart infections, heal wounds, manage inflammation, and distinguish between self and nonself. Extensive research has been undertaken to elucidate how changes in adaptive immunity contribute to such aging [1,2]. Less apparent, but growing in recognition, is the observation that changes in innate immunity have an important role in the overall decline in net immune function [3]. The innate immune system recognizes pathogens or damaged cells at the tissue–environment interface to induce local defenses, such as those against inflammation and antimicrobial peptides. These reactions also recruit and activate adaptive immunity [4]. As understanding of the mechanisms of innate immunity has developed, it has become increasingly clear that degenerative changes within this ancestral system correlate with overt symptoms of immune aging, including increased susceptibility to pathogens, chronic inflammation, and autoimmune pathology [5].

For human and animal models alike, key challenges to understanding innate immune aging are to determine the associated intrinsic and molecular changes and resolve the direction of causality between age-associated immune pathology and immune performance. Is age-associated dysregulation of immunity and inflammation a secondary response to damage because individuals are exposed to more pathogens over a longer time period? Or, is it an outcome of an age-dependent decline within the innate immune system itself, which thus contributes to chronic infection and inflammation?

*Drosophila* is an emerging model being used to address these questions in research that integrates its capacity for genetic dissection of aging with groundbreaking molecular biology related to innate immunity [6]. Explicit genetic analysis of *Drosophila* aging accelerated after Lin et al. screened for longevity assurance mutations and described the slow aging effects of *methuselah*, a GPRC [7], and after transgenic overexpression of heat shock proteins was shown to reduce age-specific mortality [8]. Subsequent to the elucidation of how mutants of the insulin/IGF signaling pathway (*daf* mutants) of *Caenorhabditis elegans* retarded aging [9], the ability of this signaling process to control aging was described for *Drosophila* [10,11]. This established the broad relevance of insulin/IGF signaling (IIS) in aging, an association that has been reinforced from mouse to human [12]. Currently, there are over 70 genes described for *Drosophila* through which genetic manipulation can increase lifespan by consistently reducing age-specific mortality, including elements of IIS (InR, IRS (insulin receptor substrate)/chico, FOXO), the TOR (target of rapamycin) pathway (TOR, 4eBP, S6K), JNK (c-Jun N-terminal kinase) signaling, autophagy, regulators of germline stem cells, detoxification, protein repair and folding, and the immune response [13]. Likewise, demographic aging can be slowed by reduced adult food intake (dietary restriction) [14], drugs (resveratrol, rapamycin) [15,16], and reducing the opportunities to mate or produce eggs [17]. Aging, as measured by adult mortality rate, is a genetically tractable *Drosophila* phenotype that is amenable to analysis of the underlying molecular mechanisms.

While most genetic analyses use demographic traits as metrics of the aging progression, research with *Drosophila* increasingly features assessment of system degeneration that models functional aging in humans. Through *Drosophila*-based research, human-related degeneration associated with declines in myocardial function, muscle, olfaction, sleep, learning/memory, stem cell maintenance, and the topic of this review—innate immunity—has been demonstrated [18]. The use of *Drosophila* as a model is especially useful when investigating innate immunity because genetic study of *Drosophila* can dissect the cause-and-effect relationship between aging and innate immune/inflammatory function. Overall, this modeling strategy is possible with *Drosophila* because the fly possesses the basic recognition and signal transduction events of mammalian innate immunity without the added complication of adaptive immunity.

## 2. Innate Immunity in *Drosophila*

### 2.1. Innate Immune Signaling in Drosophila

**Figure 1:**
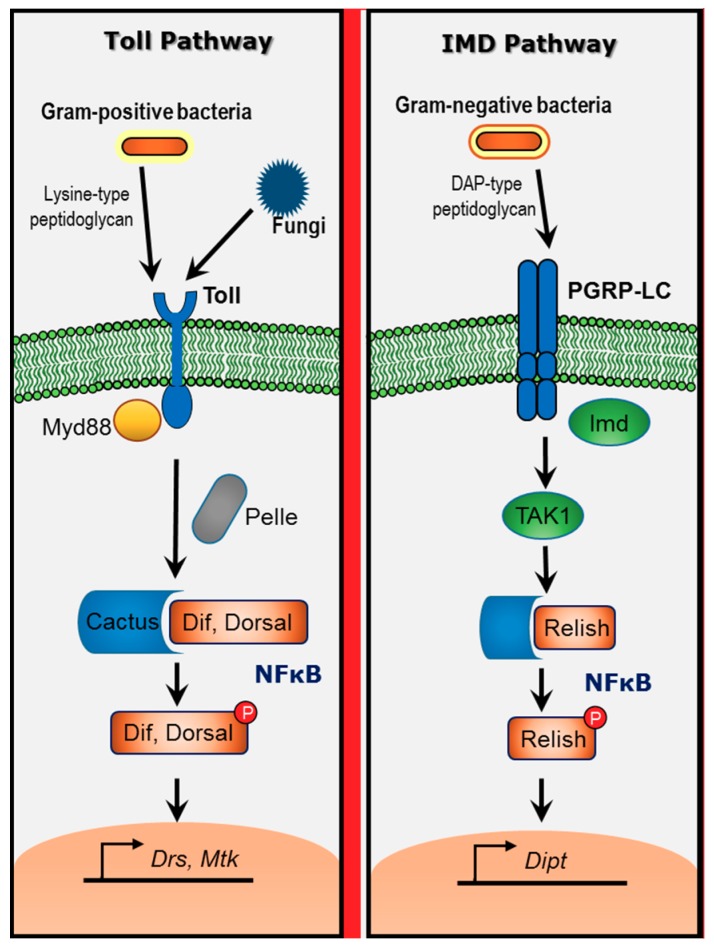
Schematic diagram of innate immune signaling in *Drosophila*. Toll pathway is activated by Gram-positive bacteria and fungi. Toll activation leads to degradation of Cactus and nuclear localization of NF-κB transcription factors Dif and Dorsal. These transcription factors induce the expression of antimicrobial genes like drosomycin (Drs) and metchnikowin (Mtk). IMD (immune deficiency) pathway is activated by Gram-negative bacteria. IMD activation leads to the nuclear translocation of NF-κB transcription factor Relish to activate the expression of antimicrobial genes like diptericin (Dipt).

When infected with microbes, *Drosophila* rapidly induces the expression of a battery of antimicrobial peptides (AMP), including diptericins, metchnikowin, defensin, cecropins, drosocin and attacins [19]. Such AMP induction is primarily controlled by the *Drosophila* homologs of NF-κB transcription factors [4]. In flies, two distinct pathways respond to microbial infection (Figure 1).

The Toll pathway recognizes lysine-type peptidoglycan, which is found in the cell wall of many Gram-positive bacteria as well as β-glucans from fungal cell walls, whereas the IMD pathway responds to DAP (diaminopimelic acid)-type peptidoglycan, from Gram-negative (and certain Gram-positive) bacterial cell walls. Once activated, the Toll and IMD pathways independently signal to specific NF-κB transcription factors. Toll activates the NF-κB homologs Dif and Dorsal, which induce the antifungal peptide genes like *drosomycin* and *metchnikowin*. The IMD pathway activates the NF-κB precursor Relish, which, among others targets, induces the antibacterial peptide gene like *diptericin*. Each of these signaling pathways is similar to the mammalian innate immune NF-κB signaling cascades. The Toll pathway is homologous to MyD88-dependent signaling downstream of most Toll-like receptors (TLRs), while the IMD pathway is more similar to the MyD88-independent (TRIF (TIR-domain-containing adapter-inducing interferon-β)-dependent) pathway downstream of TLR3 and TLR4 as well as the TNFR (tumor necrosis factor receptor) pathway [20,21,22].

### 2.2. Immune Senescence in Drosophila

A recent review by Garschall et al. described immunosenescence of *Drosophila* [23,24]. Genome expression profiling provided the first descriptions of *Drosophila* immune aging. Seroude et al. [25] reported increased messaging of antimicrobial genes, including *diptericin*, *defensin*, *attacin*, and *cecropin* as well as the peptidoglycan recognition protein (PGRP), PGRP-LC. Pletcher et al. [26] made similar observations using microarray analysis, further noting that these expression patterns were delayed in dietary restricted flies, whereas Zerofsky et al. showed an endogenous increase in mRNA of AMP among untreated, aging *Drosophila* [27]. Landis et al. reported similar patterns and also showed that high levels of *drosomycin* and *metchnikowin* in young adults predicted high mortality rates at a later age [28]. Together, these observations define the phenotype of innate immune aging in *Drosophila*: a progressive increase of AMP mRNA expression with age. However, it should be noted that there is a lack of data showing AMP peptide abundance with age because there are no effective antibodies for these peptides. Moreover, because expression patterns could be delayed by a manipulation that slows the aging process—dietary restriction (DR)—these observations support the view that innate immune aging is caused by a progressive degenerative pathology within the innate immune system.

To understand if changes in AMP expression actually reflect intrinsic age-dependent pathology, it is useful to consider alternative interpretations. In particular, aged flies may simply accumulate microbes over their lifespan and express more AMP in response. This may occur without changes in the underlying capacity or efficiency of innate immunity. The elevation in AMP expression may be an adaptive, compensatory response, and yet—as may occur in humans—such an increase in an acute innate immune response could entail a trade-off by producing chronic damage that elevates frailty. Finally, old flies may exhibit an intrinsic decline in their immune capacity as well as the cumulative effects of microbe exposure. The central challenge and potential utility of immune-related aging research with *Drosophila* is to distinguish the contribution of these potential causes, to resolve the directionality of their impacts, and to pinpoint the key underlying molecular processes.

### 2.3. Microbial Load Increase with Age

Increased microbial colonization with age appears to increase AMP expression in old *Drosophila*, and the causal relationship was tested using axenic and/or antibiotic treated flies. Ren et al. documented elevated abundance of internal and external anaerobic and aerobic bacteria in adult *Drosophila* males maintained under standard rearing conditions [29]. Axenic culture and antibiotic treatments reduced the microbial load at all ages and suppressed age-dependent increases in several, but not all, AMP mRNA levels. In contrast, Sarup et al. reported that flies reared on medium containing antibiotics and antifungal substances showed an upregulation of immune response genes with age [30]. More recently, Guo et al. documented AMP and innate immune gene expressions in intestines of aging *Drosophila* [31]. While axenic flies produced less age-dependent expression of oxidative stress-related genes and less activation of the DUOX (dual oxidase) system, AMP-related gene expression was still present, and it robustly increased with age. 

An accumulating microbe load, therefore, appears to be sufficient to increase the entire animal AMP mRNA expression level with age; however, when examined at a tissue-specific level, age-dependent induction of the IMD/rel pathway can occur in the absence of bacteria. These contrasting observations require us to refine our questions: Does microbial load increase with age because innate immune capacity is compromised? Do old adults express more AMP because they cannot manage infection, perhaps because they cannot produce functional AMP peptides (despite gene expression) or because other, nonhumoral aspects of immunity are compromised?

To address this issue, we must measure innate immune capacity independent of the current infection load. How flies respond to an acute immune challenge provides one approach to elucidation of the intrinsic mechanisms of age-related AMP abundance. Libert et al. injected adults of different ages with live *Pseudomonas aeruginosa* [32]. This controlled challenge immediately reduced survival, but there was no difference in mortality rates between adults aged seven days and 40 days. Using the same pathogen and challenge technique, Burger et al. [33] reported that DR improved postinfection survival in old adults but not in young adults. In addition, Ramsden et al. [34] studied adult survival when the adults were injected with nonpathogenic DH5 *Escherichia coli*. Survival after high-density, but not low-density, injection was reduced in aged flies. Acute mortality after injecting bacteria is sometimes, but not always, elevated in old flies, indicating that the intrinsic capacity of the innate immune system may be compromised with age.

A related approach has been used to measure the ability of aged flies to clear bacteria. For instance, one study found adults aged less than 30 days strongly suppressed injected bacteria, eliminating most bacteria within 48–72 h postinfection, whereas bacterial clearance was incomplete in males 40 days old [34]. Kim et al. reported a similar pattern in which *E. coli* clearance was ineffective in very old flies [35]. The importance of the genetic background of *Drosophila* was emphasized in an analysis of 25 wild-derived chromosome II lines, which documented considerable genotype-by-age variation in clearance [36]. Clearance was seen to decrease, to remain constant, or even to increase with age in different genotypes. Overall, and perhaps because survival is a complex trait integrated over many processes, the age-related patterns of bacterial clearance and infection survivorship do not consistently track the ubiquitous increase in AMP messaging.

Acute bacterial infection can also be used to compare the kinetics of AMP expression in old and young adult *Drosophila*. Zerofsky et al. reported on the kinetics of *diptericin* expression following injection (jabbing) with *E. coli* and *Micrococcus luteus* [27]. Compared to young flies, old adults produced a higher and longer peak of *diptericin* expression. This outcome could arise for several reasons: The innate immune system of old flies was actually more sensitive, the innate immune signaling of old flies had defective negative feedback, or the introduced bacteria proliferated more effectively in old flies. To distinguish among these alternatives, aged flies were jabbed with heat-killed (noninfective) bacteria. In this case, *diptericin* mRNA kinetics was reduced in aged adults compared to that in young adults. The intrinsic capacity of the innate immune system appears to decline with age, and this may accordingly permit introduced bacteria to more readily proliferate in aged adults and thus to elicit greater AMP expression in older animals. From this viewpoint, immune functional capacity and bacteria interact to produce the aging phenotype of age-elevated AMP mRNA expression.

### 2.4. Increased Amp Production and Aging: Consequence or Cause?

As mentioned above, a consistent observation across many studies is that AMP mRNA increases with age in normally cultured flies. Elucidation of how and why this occurs is unclear and is an area of active investigation. One factor appears to be infection history, since reducing or eliminating bacterial load represses the age-related increase in AMP mRNA [29]. There are few consistent results, however, that address an important and nonexclusive alternative: Are intrinsic processes important for the proper control of AMP decline with age? In addition, there is no clear answer as to whether the increased AMP production with aging contributes to the aging process or whether the increased AMP production with aging is a consequence of the aging process.

Results of several studies support the view that increased AMP production with aging contributes to aging. Ectopic expression of the peptidoglycan receptors PGRP-LC and PGRP-LE have been shown to reduce lifespan when begun at a young age [32,37], even though induction of PGRP-LE protected adults against introduced *P. aeruginosa* [32]. In contrast, when tested in aged flies (40 days old), PGRP-LE overexpression had no effect on the remaining life expectancy of uninfected adults while still providing an increased level of protection against acute *P. aeruginosa* infection [32]. Thus, at an age when endogenous induction of AMP is already elevated, additional activation, acutely generated, can provide some benefit against infection; however, the deleterious effects of further innate immune system induction are limited. Recently, Badinloo et al. showed that expression of Relish and AMP increase during the aging process, and overexpression of *Relish* increases AMP expression levels and decreases the lifespan in *Drosophila* [38]. The authors also reported that overexpression of individual AMPs like *attacin A*, *defensin*, *metchnikowin*, and *cecropin A1* shortened the lifespan of flies. Similarly, mild downregulation of the IMD pathway or AMP downregulation of the IMD pathway extended the lifespan of flies [39]. In contrast, overexpression of *drosocin* increased fly lifespan, possibly by reducing bacterial challenge [40]. It is unclear why there are differential effects of AMPs on fly lifespan, but it is plausible that each AMP has a different antimicrobial activity, thereby causing different changes in microbiota. Activating AMPs in axenic condition will provide a clear answer to this question.

## 3. Possible Mechanisms of Immunosenescence in *Drosophila*

### 3.1. Changes in Insulin/IGF-1 Signaling

Mutations that reduce or alter IIS extend *Drosophila* lifespan, as has been reported in other model systems such as *C. elegans* [9]. Longevity is improved in mutants of the tyrosine kinase receptor InR [41] and its insulin receptor substrate [11], in flies that produce less insulin-like peptide (Ilps) [42,43] as well as by increasing the expression of circulating Ilps-binding proteins [44]. Such genetic manipulations can postpone or retard the age-dependent degeneration of tissues and functions, such as cardiac performance and climbing ability [45]. Together, these observations suggest that IIS modulates an underlying process of senescence that can affect morbidity and pathology related to age-dependent mortality. It is potentially informative, therefore, to determine how such manipulations affect the age-related function and expression of innate immunity.

Available data related to this question are few. Libert et al. studied the effect of longevity-extending manipulations on survival after bacterial injection [46]. They found DR, which represses IIS and delays the age-dependent expression of AMPs, did not affect postinfection survival in young (10 days old) adults. Burger et al. reported that DR did not affect survival after an injection of *P. aeruginosa* in adults aged up to 21 days, but DR did improve acute survival after bacterial infection in adults older than 34 days [33]. With regard to longevity mutants, Libert et al. found that longevity assurance mutants with activated Jun-Kinase (*puc*) and IRS *chico* improved the postinfection survival of young adults (however, their effect on realized immunity with age was not reported) [46]. Strikingly, improved survivals of young *chico* and *puc* mutants were not correlated with elevated expressions of the measured AMPs, suggesting these mutants confer acute resistance either by activating aspects of nonhumoral defenses or by modulating the expression of AMP at times beyond the window when these mRNA levels were measured. Relatedly, McCormack et al. recently reported that there are elevated levels of melanization and phenoloxidase activity, but no difference in AMP gene transcripts and phagocytosis rates, in *chico* mutants [47].

**Figure 2:**
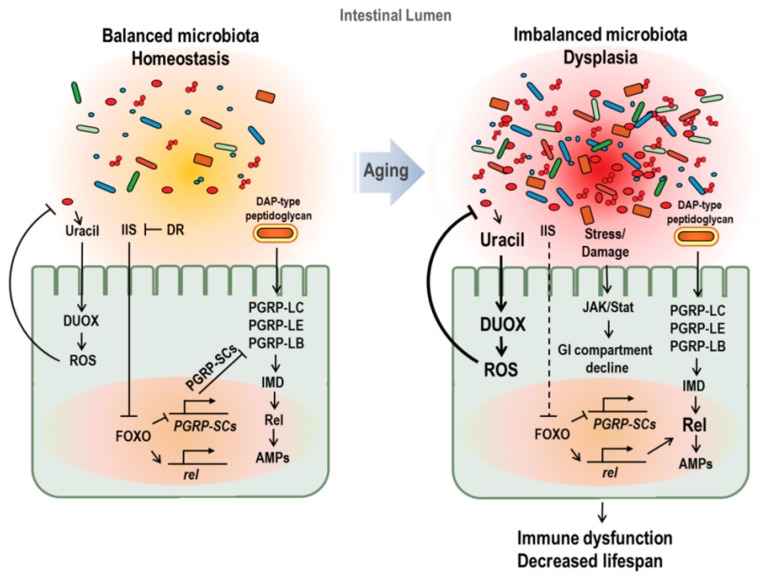
Age-dependent changes of gut microbiota and its effect on aging in *Drosophila*. In young flies (**left figure**), peptidoglycan recognition protein SC2 (PGRP-SC2) is active, facilitating the gut microbiota balance and immune homeostasis. In aged flies (**right figure**), imbalanced microbiota and dysplasia with increase in microbial loads are observed. Increased microbial load with age enriches peptidoglycans in lumen and causes a chronic inflammation. In addition, activated FOXO, caused by insulin resistance and stress accumulation, represses the PGRP-SC2 expression and enhances Relish and AMPs gene expression. Furthermore, imbalanced microbiota activates DUOX with the increase pathobionts-derived uracil. These changes induced by imbalanced microbiota in aged fly cause immune dysfunction and lifespan reduction.

A complementary approach to elucidating the potential role of IIS in innate immune aging is investigating the FOXO (forkhead box O) transcription factor, which is activated by reduced insulin signaling. FOXO is required for reduced IIS and extension of the lifespan in both *C. elegans* (via *daf-16*) and *Drosophila* [9,48]. In particular, Becker et al. showed that dFOXO directly binds to the regulatory regions of *drosomycin*, inducing its expression [49]. Based on this observation, elevation of AMP with age could result from a systemic increase in FOXO activation (Figure 2).

In fact, systemic loss of FOXO or specific loss of FOXO in enterocytes reduced age-related increases in *relish* and *diptericin* expressions [31]. However, a similar trial in our laboratory produced different results: mRNA of *drosocin* and *diptericin* increased three- and ninefold, respectively, with age (6 days versus 36 days) in *foxo*-null mutants, which is the same magnitude of change we observed in age-matched wild-type controls [50]. Furthermore, it remains unclear whether IIS signaling and FOXO activation normally decline with age in *Drosophila*, or actually increase in aged adults, although FOXO target genes and the *thor-lacZ* reporter were upregulated in aged fly intestine [31]. 

### 3.2. Hormones (JH, 20E)

Several lines of evidence suggest that juvenile hormone (JH) is a pro-aging hormone. Most of that evidence is based on *Drosophila* studies [51]. Wild-type *D. melanogaster* in diapause show downregulated levels of JH and negligible senescence [10,52], and treatment of a JH analog to dormant *Drosophila* has been shown to increase demographic senescence [41]. Several hypomorphic insulin receptor mutants of *D. melanogaster* have been shown to be long-lived. Concomitantly, the synthesis rate of JH is reduced in long-lived mutants, and treatment with a JH analog was reported to abolish the effect of *InR* mutation on longer lifespan [41]. A similar reduction of JH biosynthesis was observed in another long-lived IIS mutant, *chico* [53]. Direct evidence of the role of JH in aging was recently obtained by genetic ablation of the corpora allata, which is the JH-synthesizing organ in *Drosophila*. Corpora allata knockout flies have an increased lifespan, and treatment with a JH analog was shown to restore their lifespan to that of control flies [54]. Although fewer studies have been performed compared to those for JH, 20-hyroxyecdysone (20E) also seems to be a pro-aging hormone. Steroid hormone-deficient *molting defective (mld)-3* mutants are long-lived [55]. Several EcR heterozygote mutants are long-lived [55], and mild adult-specific EcR inactivation increases the lifespan of male *Drosophila* [56]. Interestingly, mild adult-specific EcR inactivation decreases the lifespan of females and a similar effect has been observed with strong *EcRi* and *EcR*-dominant negative isoforms in males, suggesting sex-specific control of lifespan by 20E signaling in *Drosophila* [56].

Both JH and 20E regulate immune function in *Drosophila*. The 20E hormone may reduce immune function in *Drosophila* larvae. Toll ligand-encoding gene *dorsal*, key Toll effector gene *spatzle*, and several AMP genes have been shown to be downregulated by 20E at the onset of metamorphosis in an EcR-dependent manner in a genome-wide microarray study in *Drosophila* [57]. Similarly, 20E treatment has inhibitory effects on AMP expression and activities at the final larval molt and prepupal stages in Bombyx fat bodies [58]. However, several *Drosophila* studies have suggested that 20E may act as an immune activator in adults, inducing expression of AMPs. Induction ability of the *diptericin* gene is temporally correlated with 20E, and inducibility is severely reduced in ecdysone mutants [59]. In particular, 20E indirectly modulates IMD innate immune signaling by induction of the pattern recognition receptor PGRP-LC as well as by regulation of a subset of AMP genes [60]. Therefore, 20E seems to function either as an immunosuppressor or an immunoactivator, depending on the developmental stage.

The results of many studies support the role of JH as an immune suppressor in *Drosophila*. JH treatment was shown to suppress basal expression of AMPs in microarray analysis, and JH/JH analog treatments were shown to reduce expression of *drosomycin* in vivo in *Drosophila* [61]. Similarly, application of JH I was shown to suppress synthesis of granular phenoloxidase, a key enzyme in the melanization response against pathogens, in tobacco hornworm, *Manduca sexta*. Likewise, injection of JH III was shown to suppress phenoloxidase activity and encapsulation in mealworm beetle, *Tenebrio molitor* [62]. In honeybee, *Apis mellifera*, the transition of nurse tasks to foraging tasks was associated with an increased JH titer and a marked reduction in the number of functioning hemocytes [63]. JH and 20E seem to antagonistically regulate AMP synthesis. JH III was shown to suppress transcription of AMP expression in vivo. 20E pretreatment of S2 cells was shown to increase induction of AMP [61]. However, co-treatment with JH III or JH analog was shown to interfere with 20E-induced AMP expression [61].

Considering that JH and 20E regulate both immunity and aging in *Drosophila*, it is reasonable to suggest that those hormones are involved in immunosenescence. JH and 20E production and titer have been well-characterized from larval to early adult stages, but changes in hormonal production and titer with age remain incompletely described. JH production in Canton-S flies was shown to peak on day 2 and decline up to day 10 [53]. 20E titer peaks present on the day of adult eclosion were shown to decline on day 1 and fluctuate up to day 14 [64], while recent results show that 20E titer increases with age [65]. Such age-related hormonal changes will sensitize the immune response, similar to the hyper-responsive immune response in aged flies described above, and induce chronic inflammation status upon an increased microbial load with age.

Study of autonomous AMP expression in renal Malpighian tubules has attempted to evaluate how efficiently an aged tissue can induce humoral immunity. Surprisingly, Malpighian tubules of old flies have greater potential to induce AMP expression when challenged in a controlled setting. This condition was observed to arise because water-stress dehydration induces Malpighian tubule expression of PGRP-LC, thus, priming the tubules for AMP expression. Aged flies, apparently, are normally prone to water-stress dehydration [65]. Elevated AMP expression in aged flies, therefore, may represent a by-product of a physiological adaption selected for in young flies in response to water stress; however, it may be constitutively induced and even potentially detrimental to old flies as they lose their capacity to maintain water balance.

### 3.3. Degeneration of Feedback System

The presence of commensal gut bacteria continuously activates the immune response, but prolonged immune activation is detrimental to host fitness [27]. Therefore, the host needs to suppress the immune response to commensal bacteria unless it is required, as in the event of a pathogen infection. Several positive/negative regulators have been identified, and details of their positive/negative regulation have been reviewed [66]. We propose that degenerative regulation of the immune response is one of the causes of immunosenescence.

For example, p38 MAP (mitogen activated) kinase is a stress-activated protein kinase involved in regulation of the immune response [67] and modulation of longevity [68] in *Drosophila*. Results of microarray and immunoblot analyses suggest that p38 MAP kinase activity declines with aging, which may underlie the increased susceptibility to infection observed in *C. elegans* [69]. Age-related changes in p38 MAP kinase signaling in *Drosophila* have not been characterized and require future investigation.

MicroRNAs are small noncoding RNAs that control the expression of genes at the post-transcriptional level through targeted binding to specific mRNAs. Recently, an active role of microRNAs in aging has become evident [70], and they are suggested to be involved in immunosenescence [71]. In vertebrates, mice deficient in bic/miR-155 exhibit defective adaptive immunity, and bic/miR-155 is required for functional T and B lymphocytes [72]. miR-181, an important regulator of B lymphopoiesis, shows reduced expression in peripheral blood of aged individuals [73].

Likewise, microRNAs modulate both aging and the immune response and, thus, may be an immunosenescence modulator in *Drosophila* [74]. miR-34 expression level declines with age, and miR-34 mutants exhibit age-associated defects in later life, such as reduced climbing ability, reduced lifespan, and brain degeneration [75]. Several microRNAs involved in the immune response have been identified through a screening process [76]. miR-8 regulates immune homeostasis, maintaining low expression of the AMPs *drosomycin* and *diptericin* in noninfected flies [77]. In *miR-8*-null flies, levels of *drosomycin* and *diptericin* were shown to be significantly increased without a pathogenic challenge. 3’-UTR (untranslated region) of AMP *diptericin* contains a *let-7* binding site, and binding of let-7 represses translation of *diptericin*. Expression of let-*7* is modulated by 20E, which is known to induce expression of *diptericin* [78]. Therefore, 20E works as a dual modulator of innate immunity by activating an initial immune response while also diminishing the response via microRNA. It is assumed that let-*7* sets a threshold point for AMP production, reducing overstimulation of the immune response. Regardless, direct evidence of microRNA involvement in immunosenescence is lacking, and the topic needs future investigation.

## 4. Emerging Role of Microbiota in the Regulation of Immunity and Aging in *Drosophila*

There are approximately 5–20 bacterial species in the fly gut, mostly dominated by Proteobacteria (mainly Acetobacteraceae and Enterobacteriaceae) and Firmicutes (mainly *Lactobacillus* and *Enterococcus* species), although the composition varies by research laboratory [79,80]. The gut microbiota composition can even vary temporally within the same laboratory. For example, colonization of specific gut bacteria was shown to be variable across a fly generation; *Lactobacillus brevis* and *Lactobacillus plantarum* were shown to be absent from the gut after three months [81].

### 4.1. Effect of Commensal Bacteria on Development and Host Resistance

The effects of *Acetobacter* and *Lactobacillus* on development and host resistance have been described [82,83]. A recent study by Blum et al. showed that the survival of pathogen-infected flies could be increased by the presence of the commensal bacteria *L. plantarum*. Increased survival was observed in both germ-free and conventionally reared flies, although flies with a normal microbiome were less susceptible to infection than germ-free flies [80]. A tempting explanation is that commensal bacteria can increase host resistance through increased production of reactive oxygen species (ROS) and AMP.

### 4.2. Effect of Gut Microbiota on Lifespan

There have been contrasting results obtained in studies regarding the effects of gut microbiota on the lifespan of *Drosophila*. Brummel et al. observed that experimental manipulation of microbes by axenic treatment decreased the survival of flies, whereas readdition of bacteria during the first week of the adult stage increased survival [84]. However, Ren et al. later observed that axenic or antibiotic treatments reduced microbial load with age but did not affect lifespan [29]. More recently, Ridley et al. found that the lifespan of male flies was not different between conventionally reared flies and axenic flies [85], and Clark et al. reported lifespan extension upon antibiotic treatment [24,86]. Different culture conditions could explain the differences between the results. For example, Brummel et al. reared flies on a sucrose diet, whereas Ren et al. reared flies on a dextrose diet. The identity and composition of commensal bacteria can greatly vary with different diets. Young adult flies contained *Acetobacter* and *Lactobacillus* at a ratio of 49:1 when reared on a diet containing 4.8% yeast, but the ratio was reversed to 1:4 on a diet containing 8.6% yeast, even though both studies were performed in the same laboratory [78,85]. A recent report showed that host–microbe interactions are complex and nutrient-dependent. Yamada et al. observed that microbes are involved in amino acid harvest and extension of lifespan under malnutrition [87]. Therefore, gut-associated microbes may have different effects on a host’s lifespan depending on nutrient condition. Another possibility is that differences in residing microbial species and different alterations in microbial composition associated with aging can produce differential responses to axenic conditions among laboratories.

Recent results support the assertion that increased microbial challenge with aging is a key determinant of the *Drosophila* lifespan. Gould et al. found that germ-free flies live longer than conventional flies and that a decrease in survival is a function of bacterial load [88]. Loch et al. also observed that overexpression of *drosocin* increased the lifespan of flies and that a reduction of bacterial challenge upon *drosocin* expression may be responsible for the longer lifespan [40].

### 4.3. Age-Dependent Changes in Gut Bacteria Populations

Brummel et al. observed that, contrary to the beneficial effect of early bacterial exposure, removal of bacteria in later life stages can increase fly survival [84]. Thus, although the presence of bacteria in early life may be beneficial, their presence in later life may be detrimental. This leads to questions about gut composition changes with age. Wong et al. reported that *L. fructivorans* is the most abundant species in young adult flies, whereas *A. pomorum* is the most abundant in older adults [79]. In contrast, Ridley et al. observed that *A. pomorum* is the most abundant in young adults [85], whereas Ryu et al. reported that *L. plantarum* and an Acetobacteracean bacterium—strain EW911 (A911)—were the predominant species in young adults [89]. Blum et al. reported that two major species—*Lactobacillus* and *Acetobacter*—comprising 94% of the microbiome, dominated the bacterial population of flies from day 2 to day 54 [80]. More recently, Han et al. performed a comparative analysis of gut microbiota in *w^1118^* and Canton-S fly strains. The relative proportions of the major bacterial genera—*Lactobacillus*, *Acetobacter*, *Enterococcus*, and *Leuconostoc*—were significantly different with respect to host age, sex, and strain [90]. Therefore, there are no universal bacteria specific to young stage in the fly gut, and the identities of bacteria in the adult gut needs more investigation.

*Gluconobacter morbifer* comprises a minor proportion of the gut of wild-type flies, whereas it is dominant in immune-deficient *caudal* RNAi flies. Gut epithelial apoptosis and host mortality have been shown to be increased in germ-free flies when *G. morbifer* was singly introduced [89]. Thus, the population dynamic and role of *G. morbifer* during normal aging require further investigation.

### 4.4. Changes in Intestinal Immunity and Physiology by Age

Protection against microbial infection involves several barriers, including AMP secretion and production of ROS in intestinal epithelium. Peptidoglycan in bacteria activates the Relish-dependent IMD signaling pathway and boosts production of AMP [91]. DUOX induces generation of ROS, controlling gut bacteria proliferation and homeostasis. A recent study by Lee et al. showed that bacterial uracil is an elicitor of DUOX. Symbionts do not produce uracil without DUOX activation, but pathobionts such as *G. morbifer* and *L. brevis* release uracil, inducing chronic inflammation status and host death [92].

Unlike uracil release by pathobionts only, peptidoglycan is released from most gut bacteria. An increase in microbial load with age creates a peptidoglycan-abundant environment and induces constitutive AMP production, causing chronic inflammation status [93]. Aging also regulates negative regulators of IMD/relish-dependent AMP production. In young flies, peptidoglycan recognition protein SC2 (PGRP-SC2), a negative regulator of the IMD/relish signaling pathway, is active and helps maintain immune homeostasis. In aged flies, activated FOXO, presumably by insulin resistance and/or stress accumulation, represses expression of PGRP-SC2, inducing chronic AMP production, commensal community changes, epithelial dysplasia, and host mortality [31].

Similar to lamin B1 loss and the senescence-associated secretory phenotype in aged mammalian fibroblasts, *Drosophila* exhibit age-associated lamin-B loss and systematic inflammation. Lamin-B is gradually lost in fat bodies with age, resulting in systematic inflammation. Inflamed old fat bodies secrete PGRPs which repress IMD in the midgut and induce gut hyperplasia [94].

It has been proposed that AMP-resistant pathobionts such as *G. morbifer* and *L. brevis* dominate in the gut of aged flies, resulting in gut dysplasia and a shortened lifespan in *Drosophila* [95]. However, there has been no direct evidence of this until recently (see Section 4.3 Age-dependent changes in gut bacteria populations). Clark et al. observed that a distinct shift in microbial composition—increase in Gammaproteobacteria and decrease in Firmicutes—precedes intestinal barrier dysfunction [86]. This age-dependent loss of commensal control induces systemic immune activation and drives mortality in *Drosophila*.

The intestinal epithelium experiences constant renewal of cells provided by intestinal stem cells (ISCs). Dysregulation of ISC proliferation in aged *Drosophila* intestine results in epithelial dysplasia, causing leakage of epithelial barrier, systemic infection, metabolic dysregulation and death in animals [96,97,98]. Similarly, manipulation that limits the rate of ISC proliferation in the aging intestine is sufficient to extend lifespan in *Drosophila* [31,96]. ISC proliferation rates are regulated by numerous signaling pathways, including Notch, JAK (janus kinase)/Stat, insulin, JNK, and TOR and by environmental conditions [99]. Recently, it was found that intermittent fasting during early life not only extended the lifespan of flies, but also preserved the gut homeostasis [100]. The impact of diet on ISC proliferation was also compared between *Drosophila* females and males. The study found DR reduced gut pathology more in aging females than in aging males, which can possibly be attributed to greater response to DR in females [101].

## 5. Conclusions

While an elevation in AMP mRNA expression with age was initially described as a feature of innate immune senescence in *Drosophila*, cumulative insights since that time suggest that the increase in AMP expression in old animals is an epiphenomenon. Old animals express more AMP because they have greater bacterial loads and because the humoral innate immune system is more sensitive to a set amount of bacterial stimulation. Many factors contribute to this former cause, such as the intrinsic degeneration of nonhumoral immune defenses including the gut barrier, DUOX, and hemocyte cell function. When these initial defenses decline with age, bacteria may be more likely to proliferate in the hemolymph of adult *Drosophila* and, thereby, induce systemic humoral AMP expression. At the same time, the humoral innate immune system may still degenerate in old adults, synergistically contributing to the elevated microbial load and eventually, although inefficiently, inducing AMP expression. 

## Abbreviations

IIS	insulin/IGF signaling
AMP	antimicrobial peptides
DR	dietary restriction
20E	20-hydroxyecdysone
JH	juvenile Hormone
FOXO	forkhead box O
PGRP	peptidoglycan recognition protein
DUOX	dual oxidase

## References

[1] Miller R.A. (1996). The aging immune system: Primer and prospectus. Science.

[2] Weng N.P. (2006). Aging of the immune system: How much can the adaptive immune system adapt?. Immunity.

[3] Solana R., Pawelec G., Tarazona R. (2006). Aging and innate immunity. Immunity.

[4] Silverman N., Maniatis T. (2001). NF-κB signaling pathways in mammalian and insect innate immunity. Genes Dev..

[5] Renshaw M., Rockwell J., Engleman C., Gewirtz A., Katz J., Sambhara S. (2002). Cutting edge: Impaired Toll-like receptor expression and function in aging. J. Immunol..

[6] Eleftherianos I., Castillo J.C. (2012). Molecular mechanisms of aging and immune system regulation in *Drosophila*. Int. J. Mol. Sci..

[7] Lin Y.J., Seroude L., Benzer S. (1998). Extended life-span and stress resistance in the *Drosophila* mutant *methuselah*. Science.

[8] Tatar M., Khazaeli A.A., Curtsinger J.W. (1997). Chaperoning extended life. Nature.

[9] Kenyon C., Chang J., Gensch E., Rudner A., Tabtiang R.A. (1993). *C. elegans* mutant that lives twice as long as wild type. Nature.

[10] Tatar M., Chien S.A., Priest N.K. (2001). Negligible Senescence during Reproductive Dormancy in *Drosophila* melanogaster. Am. Nat..

[11] Clancy D.J., Gems D., Harshman L.G., Oldham S., Stocker H., Hafen E., Leevers S.J., Partridge L. (2001). Extension of life-span by loss of CHICO, a *Drosophila* insulin receptor substrate protein. Science.

[12] Tatar M., Bartke A., Antebi A. (2003). The endocrine regulation of aging by insulin-like signals. Science.

[13] Proshkina E.N., Shaposhnikov M.V., Sadritdinova A.F., Kudryavtseva A.V., Moskalev A.A. (2015). Basic mechanisms of longevity: A case study of *Drosophila* pro-longevity genes. Ageing Res. Rev..

[14] Mair W., Goymer P., Pletcher S.D., Partridge L. (2003). Demography of dietary restriction and death in *Drosophila*. Science.

[15] Bjedov I., Toivonen J.M., Kerr F., Slack C., Jacobson J., Foley A., Partridge L. (2010). Mechanisms of life span extension by rapamycin in the fruit fly *Drosophila melanogaster*. Cell Metab..

[16] Wood J.G., Rogina B., Lavu S., Howitz K., Helfand S.L., Tatar M., Sinclair D. (2004). Sirtuin activators mimic caloric restriction and delay ageing in metazoans. Nature.

[17] Harshman L.G., Zera A.J. (2007). The cost of reproduction: The devil in the details. Trends Ecol. Evol..

[18] Lee H.Y., Lee S.H., Min K.J. (2014). Insects as a model system for aging studies. Entomol. Res..

[19] Hetru C., Troxler L., Hoffmann J.A. (2003). *Drosophila melanogaster* antimicrobial defense. J. Infect. Dis..

[20] Fitzgerald K.A., Rowe D.C., Barnes B.J., Caffrey D.R., Visintin A., Latz E., Monks B., Pitha P.M., Golenbock D.T. (2003). LPS-TLR4 signaling to IRF-3/7 and NF-**κ**B involves the toll adapters TRAM and TRIF. J. Exp. Med..

[21] Meylan E., Burns K., Hofmann K., Blancheteau V., Martinon F., Kelliher M., Tschopp J. (2004). RIP1 is an essential mediator of Toll-like receptor 3-induced NF-κB. activation. Nat. Immunol..

[22] Takeda K., Akira S. (2005). Toll-like receptors in innate immunity. Int. Immunol..

[23] Garschall K., Flatt T. (2018). The interplay between immunity and aging in *Drosophila*. F1000Research.

[24] Clark R.I., Walker D.W. (2018). Role of gut microbiota in aging-related health decline: Insights from invertebrate models. Cell. Mol. Life Sci..

[25] Seroude L., Brummel T., Kapahi P., Benzer S. (2002). Spatio-temporal analysis of gene expression during aging in *Drosophila melanogaster*. Aging Cell.

[26] Pletcher S.D., Macdonald S.J., Marguerie R., Certa U., Stearns S.C., Goldstein D.B., Partridge L. (2002). Genome-wide transcript profiles in aging and calorically restricted *Drosophila melanogaster*. Curr. Biol..

[27] Zerofsky M., Harel E., Silverman N., Tatar M. (2005). Aging of the innate immune response in *Drosophila melanogaster*. Aging Cell.

[28] Landis G.N., Abdueva D., Skvortsov D., Yang J., Rabin B.E., Carrick J., Tavare S., Tower J. (2004). Similar gene expression patterns characterize aging and oxidative stress in *Drosophila melanogaster*. Proc. Natl. Acad. Sci. USA.

[29] Ren C., Webster P., Finkel S.E., Tower J. (2007). Increased internal and external bacterial load during *Drosophila* aging without life-span trade-off. Cell Metab..

[30] Sarup P., Sorensen P., Loeschcke V. (2011). Flies selected for longevity retain a young gene expression profile. Age (Dordr).

[31] Guo L., Karpac J., Tran S.L., Jasper H. (2014). PGRP-SC2 promotes gut immune homeostasis to limit commensal dysbiosis and extend lifespan. Cell.

[32] Libert S., Chao Y., Chu X., Pletcher S.D. (2006). Trade-offs between longevity and pathogen resistance in *Drosophila melanogaster* are mediated by NF-κB signaling. Aging Cell.

[33] Burger J.M., Hwangbo D.S., Corby-Harris V., Promislow D.E. (2007). The functional costs and benefits of dietary restriction in *Drosophila*. Aging Cell.

[34] Ramsden S., Cheung Y.Y., Seroude L. (2008). Functional analysis of the *Drosophila* immune response during aging. Aging Cell.

[35] Kim Y.S., Nam H.J., Chung H.Y., Kim N.D., Ryu J.H., Lee W.J., Arking R., Yoo M.A. (2001). Role of xanthine dehydrogenase and aging on the innate immune response of *Drosophila*. J. Am. Aging Assoc..

[36] Lesser K.J., Paiusi I.C., Leips J. (2006). Naturally occurring genetic variation in the age-specific immune response of *Drosophila melanogaster*. Aging Cell.

[37] DeVeale B., Brummel T., Seroude L. (2004). Immunity and aging: The enemy within?. Aging Cell.

[38] Badinloo M., Nguyen E., Suh W., Alzahrani F., Castellanos J., Klichko V.I., Orr W.C., Radyuk S.N. (2018). Overexpression of antimicrobial peptides contributes to aging through cytotoxic effects in *Drosophila* tissues. Arch. Insect. Biochem. Physiol..

[39] Lin Y.R., Parikh H., Park Y. (2018). Stress resistance and lifespan enhanced by downregulation of antimicrobial peptide genes in the Imd pathway. Aging (Albany NY).

[40] Loch G., Zinke I., Mori T., Carrera P., Schroer J., Takeyama H., Hoch M. (2017). Antimicrobial peptides extend lifespan in *Drosophila*. PLoS ONE.

[41] Tatar M., Kopelman A., Epstein D., Tu M.P., Yin C.M., Garofalo R.S. (2001). A mutant *Drosophila* insulin receptor homolog that extends life-span and impairs neuroendocrine function. Science.

[42] Broughton S.J., Piper M.D., Ikeya T., Bass T.M., Jacobson J., Driege Y., Martinez P., Hafen E., Withers D.J., Leevers S.J. (2005). Longer lifespan, altered metabolism, and stress resistance in *Drosophila* from ablation of cells making insulin-like ligands. Proc. Natl. Acad. Sci. USA.

[43] Gronke S., Clarke D.F., Broughton S., Andrews T.D., Partridge L. (2010). Molecular evolution and functional characterization of *Drosophila* insulin-like peptides. PLoS Genet..

[44] Alic N., Hoddinott M.P., Vinti G., Partridge L. (2011). Lifespan extension by increased expression of the *Drosophila* homologue of the IGFBP7 tumour suppressor. Aging Cell.

[45] Wessells R.J., Fitzgerald E., Cypser J.R., Tatar M., Bodmer R. (2004). Insulin regulation of heart function in aging fruit flies. Nat. Genet..

[46] Libert S., Chao Y., Zwiener J., Pletcher S.D. (2008). Realized immune response is enhanced in long-lived *puc* and *chico* mutants but is unaffected by dietary restriction. Mol. Immunol..

[47] McCormack S., Yadav S., Shokal U., Kenney E., Cooper D., Eleftherianos I. (2016). The insulin receptor substrate Chico regulates antibacterial immune function in *Drosophila*. Immun. Ageing.

[48] Slack C., Giannakou M.E., Foley A., Goss M., Partridge L. (2011). dFOXO-independent effects of reduced insulin-like signaling in *Drosophila*. Aging Cell.

[49] Becker T., Loch G., Beyer M., Zinke I., Aschenbrenner A.C., Carrera P., Inhester T., Schultze J.L., Hoch M. (2010). FOXO-dependent regulation of innate immune homeostasis. Nature.

[50] Tatar M. (2018). Brown University, Providence, RI, USA.

[51] Flatt T., Tu M.P., Tatar M. (2005). Hormonal pleiotropy and the juvenile hormone regulation of *Drosophila* development and life history. Bioessays.

[52] Saunders D.S., Richard D.S., Applebaum S.W., Ma M., Gilbert L.I. (1990). Photoperiodic diapause in *Drosophila* melanogaster involves a block to the juvenile hormone regulation of ovarian maturation. Gen. Comp. Endocrinol..

[53] Tu M.P., Yin C.M., Tatar M. (2005). Mutations in insulin signaling pathway alter juvenile hormone synthesis in *Drosophila melanogaster*. Gen. Comp. Endocrinol..

[54] Yamamoto R., Bai H., Dolezal A.G., Amdam G., Tatar M. (2013). Juvenile hormone regulation of *Drosophila* aging. BMC Biol..

[55] Simon A.F., Shih C., Mack A., Benzer S. (2003). Steroid control of longevity in *Drosophila melanogaster*. Science.

[56] Tricoire H., Battisti V., Trannoy S., Lasbleiz C., Pret A.M., Monnier V. (2009). The steroid hormone receptor EcR finely modulates *Drosophila* lifespan during adulthood in a sex-specific manner. Mech. Ageing Dev..

[57] Beckstead R.B., Lam G., Thummel C.S. (2005). The genomic response to 20-hydroxyecdysone at the onset of *Drosophila metamorphosis*. Genome Biol..

[58] Tian L., Guo E., Diao Y., Zhou S., Peng Q., Cao Y., Ling E., Li S. (2010). Genome-wide regulation of innate immunity by juvenile hormone and 20-hydroxyecdysone in the *Bombyx* fat body. BMC Genom..

[59] Meister M., Richards G. (1996). Ecdysone and insect immunity: The maturation of the inducibility of the *diptericin* gene in *Drosophila* larvae. Insect Biochem. Mol. Biol..

[60] Rus F., Flatt T., Tong M., Aggarwal K., Okuda K., Kleino A., Yates E., Tatar M., Silverman N. (2013). Ecdysone triggered PGRP-LC expression controls *Drosophila* innate immunity. EMBO J..

[61] Flatt T., Heyland A., Rus F., Porpiglia E., Sherlock C., Yamamoto R., Garbuzov A., Palli S.R., Tatar M., Silverman N. (2008). Hormonal regulation of the humoral innate immune response in *Drosophila melanogaster*. J. Exp. Biol..

[62] Rantala M.J., Vainikka A., Kortet R. (2003). The role of juvenile hormone in immune function and pheromone production trade-offs: A test of the immunocompetence handicap principle. Proc. Biol. Sci..

[63] Amdam G.V., Simoes Z.L., Hagen A., Norberg K., Schroder K., Mikkelsen O., Kirkwood T.B., Omholt S.W. (2004). Hormonal control of the yolk precursor vitellogenin regulates immune function and longevity in honeybees. Exp. Gerontol..

[64] Handler A.M. (1982). Ecdysteroid titers during pupal and adult development in *Drosophila melanogaster*. Dev. Biol..

[65] Zheng W., Rus F., Hernandez A., Kang P., Goldman W., Silverman N., Tatar M. (2018). Dehydration triggers ecdysone-mediated recognition-protein priming and elevated anti-bacterial immune responses in *Drosophila* Malpighian tubule renal cells. BMC Biol..

[66] Aggarwal K., Silverman N. (2008). Positive and negative regulation of the *Drosophila* immune response. BMB Rep..

[67] Han Z.S., Enslen H., Hu X., Meng X., Wu I.H., Barrett T., Davis R.J., Ip Y.T. (1998). A conserved p38 mitogen-activated protein kinase pathway regulates *Drosophila* immunity gene expression. Mol. Cell. Biol..

[68] Vrailas-Mortimer A., del Rivero T., Mukherjee S., Nag S., Gaitanidis A., Kadas D., Consoulas C., Duttaroy A., Sanyal S. (2011). A muscle-specific p38 MAPK/Mef2/MnSOD pathway regulates stress, motor function, and life span in *Drosophila*. Dev. Cell.

[69] Youngman M.J., Rogers Z.N., Kim D.H. (2011). A decline in p38 MAPK signaling underlies immunosenescence in *Caenorhabditis elegans*. PLoS Genet..

[70] Harries L.W. (2014). MicroRNAs as Mediators of the Ageing Process. Genes.

[71] Aalaei-andabili S., Zare-Bidoki A., Rezaei N., Massoud A., Rezaei N. (2014). The role of microRNAs in immunosenescence process. Immunology of Aging.

[72] Rodriguez A., Vigorito E., Clare S., Warren M.V., Couttet P., Soond D.R., van Dongen S., Grocock R.J., Das P.P., Miska E.A. (2007). Requirement of bic/microRNA-155 for normal immune function. Science.

[73] Seeger T., Haffez F., Fischer A., Koehl U., Leistner D.M., Seeger F.H., Boon R.A., Zeiher A.M., Dimmeler S. (2013). Immunosenescence-associated microRNAs in age and heart failure. Eur. J. Heart Fail..

[74] Stanley D. (2012). Aging and immunosenescence in invertebrates. Invertebr. Surviv. J..

[75] Liu N., Landreh M., Cao K., Abe M., Hendriks G.J., Kennerdell J.R., Zhu Y., Wang L.S., Bonini N.M. (2012). The microRNA miR-34 modulates ageing and neurodegeneration in *Drosophila*. Nature.

[76] Fullaondo A., Lee S.Y. (2012). Identification of putative miRNA involved in *Drosophila melanogaster* immune response. Dev. Comp. Immunol..

[77] Choi I.K., Hyun S. (2012). Conserved microRNA miR-8 in fat body regulates innate immune homeostasis in *Drosophila*. Dev. Comp. Immunol..

[78] Garbuzov A., Tatar M. (2010). Hormonal regulation of *Drosophila* microRNA let-7 and miR-125 that target innate immunity. Fly (Austin).

[79] Wong C.N., Ng P., Douglas A.E. (2011). Low-diversity bacterial community in the gut of the fruitfly *Drosophila melanogaster*. Environ. Microbiol..

[80] Blum J.E., Fischer C.N., Miles J., Handelsman J. (2013). Frequent replenishment sustains the beneficial microbiome of *Drosophila melanogaster*. mBio.

[81] Wong A.C., Chaston J.M., Douglas A.E. (2013). The inconstant gut microbiota of *Drosophila* species revealed by 16S rRNA gene analysis. ISME J..

[82] Storelli G., Defaye A., Erkosar B., Hols P., Royet J., Leulier F. (2011). *Lactobacillus plantarum* promotes *Drosophila* systemic growth by modulating hormonal signals through TOR-dependent nutrient sensing. Cell Metab..

[83] Shin S.C., Kim S.H., You H., Kim B., Kim A.C., Lee K.A., Yoon J.H., Ryu J.H., Lee W.J. (2011). *Drosophila* microbiome modulates host developmental and metabolic homeostasis via insulin signaling. Science.

[84] Brummel T., Ching A., Seroude L., Simon A.F., Benzer S. (2004). *Drosophila* lifespan enhancement by exogenous bacteria. Proc. Natl. Acad. Sci. USA.

[85] Ridley E.V., Wong A.C., Westmiller S., Douglas A.E. (2012). Impact of the resident microbiota on the nutritional phenotype of *Drosophila melanogaster*. PLoS ONE.

[86] Clark R.I., Salazar A., Yamada R., Fitz-Gibbon S., Morselli M., Alcaraz J., Rana A., Rera M., Pellegrini M., Ja W.W. (2015). Distinct Shifts in Microbiota Composition during *Drosophila* Aging Impair Intestinal Function and Drive Mortality. Cell Rep..

[87] Yamada R., Deshpande S.A., Bruce K.D., Mak E.M., Ja W.W. (2015). Microbes Promote Amino Acid Harvest to Rescue Undernutrition in *Drosophila*. Cell Rep..

[88] Gould A.L., Zhang V., Lamberti L., Jones E.W., Obadia B., Gavryushkin A., Carlson J.M., Beerenwinkel N., Ludington W.B. (2017). High-dimensional microbiome interactions shape host fitness. BioRxiv.

[89] Ryu J.H., Kim S.H., Lee H.Y., Bai J.Y., Nam Y.D., Bae J.W., Lee D.G., Shin S.C., Ha E.M., Lee W.J. (2008). Innate immune homeostasis by the homeobox gene *caudal* and commensal-gut mutualism in *Drosophila*. Science.

[90] Han G., Lee H.J., Jeong S.E., Jeon C.O., Hyun S. (2017). Comparative Analysis of *Drosophila melanogaster* Gut Microbiota with Respect to Host Strain, Sex, and Age. Microb. Ecol..

[91] Choe K.M., Werner T., Stoven S., Hultmark D., Anderson K.V. (2002). Requirement for a peptidoglycan recognition protein (PGRP) in Relish activation and antibacterial immune responses in *Drosophila*. Science.

[92] Lee K.A., Kim S.H., Kim E.K., Ha E.M., You H., Kim B., Kim M.J., Kwon Y., Ryu J.H., Lee W.J. (2013). Bacterial-derived uracil as a modulator of mucosal immunity and gut-microbe homeostasis in *Drosophila*. Cell.

[93] Lee W.J., Hase K. (2014). Gut microbiota-generated metabolites in animal health and disease. Nat. Chem. Biol..

[94] Chen H., Zheng X., Zheng Y. (2014). Age-associated loss of lamin-B leads to systemic inflammation and gut hyperplasia. Cell.

[95] Erkosar B., Leulier F. (2014). Transient adult microbiota, gut homeostasis and longevity: Novel insights from the *Drosophila* model. FEBS Lett..

[96] Biteau B., Karpac J., Supoyo S., Degennaro M., Lehmann R., Jasper H. (2010). Lifespan extension by preserving proliferative homeostasis in *Drosophila*. PLoS Genet..

[97] Rera M., Clark R.I., Walker D.W. (2012). Intestinal barrier dysfunction links metabolic and inflammatory markers of aging to death in *Drosophila*. Proc. Natl. Acad. Sci. USA.

[98] Karpac J., Biteau B., Jasper H. (2013). Misregulation of an adaptive metabolic response contributes to the age-related disruption of lipid homeostasis in *Drosophila*. Cell Rep..

[99] Li H., Jasper H. (2016). Gastrointestinal stem cells in health and disease: From flies to humans. Dis. Model. Mech..

[100] Catterson J.H., Khericha M., Dyson M.C., Vincent A.J., Callard R., Haveron S.M., Rajasingam A., Ahmad M., Partridge L. (2018). Short-Term, Intermittent Fasting Induces Long-Lasting Gut Health and TOR-Independent Lifespan Extension. Curr. Biol..

[101] Regan J.C., Khericha M., Dobson A.J., Bolukbasi E., Rattanavirotkul N., Partridge L. (2016). Sex difference in pathology of the ageing gut mediates the greater response of female lifespan to dietary restriction. eLife.

